# Physical, Chemical, and Immunohistochemical Investigation of the Damage to Salivary Glands in a Model of Intoxication with Aluminium Citrate

**DOI:** 10.3390/ijerph111212429

**Published:** 2014-11-28

**Authors:** Natacha M. M. da Costa, Russell S. Correa, Ismael S. M. Júnior, Adilson J. R. Figueiredo, Kelly F. B. Vilhena, Paulo M. A. Farias-Junior, Francisco B. Teixeira, Nayana M. M. Ferreira, João B. Pereira-Júnior, Kelly das Graças F. Dantas, Marcia C. F. da Silva, Ademir F. Silva-Junior, Sergio de M. Alves-Junior, João de Jesus V. Pinheiro, Rafael Rodrigues Lima

**Affiliations:** 1 Laboratory of Functional and Structural Biology, Institute of Biological Sciences, Federal University of Pará, 66075-900 Belém-Pará, Brazil; E-Mails: natacha_malu@hotmail.com (N.M.M.C.); russellsantiago@hotmail.com (R.S.C.); ismaeljunior7@hotmail.com (I.S.M.J.); adilsonlogia@yahoo.com.br (A.J.R.F.); kelly.vilhena@hotmail.com (K.F.B.V.); paulo.junior@ics.ufpa.br (P.M.A.F.-J.); teixeira.f.bruno@gmail.com (F.B.T.); nayanamoraes89@hotmail.com (N.M.M.F.); marciaf@ufpa.br (M.C.F.S.); ademirjunior@ufpa.br (A.F.S.-J.); 2 School of Chemistry, Institute of Exact and Natural Sciences, Federal University of Pará, 66075-900 Belém-Pará, Brazil; E-Mails: joaobatista.junior@gmail.com (J.B.P.-J.); kdgfernandes@ufpa.br (K.G.F.D.); 3 School of Dentistry, Institute of Health Sciences, Federal University of Pará, 66075-900 Belém-Pará, Brazil; E-Mails: sergioalves6@hotmail.com (S.M.A.-J.); radface@hotmail.com (J.J.V.P.)

**Keywords:** aluminum citrate, toxicology, salivary gland

## Abstract

Aluminum absorption leads to deposits in several tissues. In this study, we have investigated, to our knowledge for the first time, aluminum deposition in the salivary glands in addition to the resultant cellular changes in the parotid and submandibular salivary glands in a model of chronic intoxication with aluminum citrate in rats. Aluminum deposits were observed in the parotid and submandibular glands. Immunohistochemical evaluation of cytokeratin-18 revealed a decreased expression in the parotid gland with no changes in the submandibular gland. A decreased expression of α-smooth muscle actin was observed in the myoepithelial cells of both glands. The expression of metallothionein I and II (MT-I/II), a group of metal-binding proteins, which are useful indicators for detecting physiological responses to metal exposure, was higher in both glands. In conclusion, we have shown that at a certain time and quantity of dosage, aluminum citrate promotes aluminum deposition in the parotid and submandibular glands, leads to an increased expression of MT-I/II in both the glands, damages the cytoskeleton of the myoepithelial cells in both glands, and damages the cytoskeleton of the acinar/ductal cells of the parotid glands, with the submandibular glands showing resistance to the toxicity of the latter.

## 1. Introduction

Aluminum (Al) is the third most common element in the Earth’s crust and the most abundant of the metals [1]. Al is found in large concentrations in the soil and in the air around waste disposal sites, especially those of industries that use coal and Al mining companies [2,3,4,5,6]. The bioavailability of Al stems mainly from acid rain, which releases Al from the soil into fresh water, making it accessible to living organisms [7]. Such environmental availability in combination with its industrial use makes Al an important environmental pollutant, and its toxicity depends directly on its chemical form [5].

The gastrointestinal tract is the main site of Al absorption in humans. Al can be released by cookware during cooking and may also be found in food, water, vitamins, and supplements. It can also be absorbed through other routes, such as through the skin and by inhalation, through the use of aerosol cosmetics, which may promote absorption by both routes, and also by the use of sunscreens, make-up, and tanning lotions. Medicines and vaccines may also contribute to Al deposition in the body [1].

Many studies have been conducted over the years on the effects of Al in the human body. It is known that this metal accumulates in bone, liver and kidney cells, and can cause a decline in liver and kidney functions, a lack of motor coordination, abdominal cramps, headaches and seizures [8,9,10,11,12]. On the other hand, little is known regarding the toxicity of Al in glandular tissue, although some evidence suggests an action of the metal in mammary glands, parathyroid glands and salivary glands [11,13,14,15,16,17,18].

Studies relating the decrease of salivary flow and the morphological alterations in salivary glands in rats after Al poisoning during 15 days have been reported [19]. Taking this into account, we considered it to be scientifically relevant to assess Al deposits, and the expression of structural proteins (cytokeratin 18, and α-actin in the smooth muscle) and metallothionein to understand the mechanism by which Al intoxication might alter the salivary glands. As far as we know, no study dealing with these questions has been conducted to date.

## 2. Methods 

### 2.1. Experimental Animals

Forty male adult Wistar rats (250–300 g) were obtained from the Federal University of Pará Central Animal Facility. All animals were housed under standard conditions (25 °C, 12 h light-dark cycle) with food and water available *ad libitum*. All experimental procedures were carried out in accordance with the Principles of Laboratory Animal Care (NIH publication No. 86–23, revised 1985) and European Commission Directive 86/609/EEC for animal experiments under license of the Ethics Committee on Experimental Animals of the Federal University of Pará.

### 2.2. Aluminum Citrate Intoxication and Experimental Groups

The animals were given a daily single dose of Al citrate (100 mg/kg/day) by gavage for 30 days. The animals were divided into two groups of twenty animals each. The control group received only H_2_O and the experimental group was exposed to Al citrate.

### 2.3. Surgical Procedures

Twenty four hours after the last treatment, the animals were deeply anesthetized with ketamine hydrochloride (72 mg/kg, intraperitoneally; i.p.) and xylazine hydrochloride (9 mg/kg, i.p.). Surgical manipulation was performed only after both the corneal and the paw withdraw reflexes were abolished. Surgery was performed to collect the parotid and submandibular salivary glands of the face side of the animal. These glands were used to analyze the concentration of Al citrate. Glands of other side were also collected for analysis after transcardial perfusion as described below.

### 2.4. Sample Digestion and Quantification of Al by Graphite Furnace Atomic Absorption Spectrometry (GF AAS)

#### 2.4.1. Instrumentation 

A lyophilizer (Model L101, Liotop, Sao Carlos, SP, Brazil) was used for drying the samples. After drying, the samples were pulverized with a mortar and pestle. Acid digestion of samples was performed in a microwave oven (Start E, Milestone, Sorisole, Italy) [20]. Determinations of the total concentration of Al in the digested samples were carried out using a Varian Spectra AA 240Z atomic absorption spectrometer (Mulgrave, Victoria, Australia) equipped with a transverse Zeeman effect background corrector and a longitudinal-heated graphite furnace atomizer. An Al hollow cathode lamp was employed as a radiation source, operating at 10 mA. Absorbance signals were measured using the 256.8 nm line at a spectral resolution of 0.5 nm. All measurements were based on integrated absorbance. Argon (99.999% purity, Linde, Pará, Brazil), was used as the purge gas. The temperature programs used for determination Al in samples are shown in Table 1.

**Table 1:** Measurement of the average levels of Al deposition in the salivary glands represented in μg·g^−1^ ± standard deviations.

Samples	(Al Levels—ppm) ± Standard Deviation
Submandibular (control)	278.7 ± 32.2
Submandibular exposed to Al	397.3 ± 15.3
Parotid (control)	202.2 ± 0.1
Parotid exposed to Al	274.9 ± 0.1

#### 2.4.2. Reagents and Solutions

All reagents used were analytical grade. All dilutions were made using distilled-deionized water (resistivity 18.2 MΩ cm) obtained from an ELGA water purification system (Elgastat, Buckinghamshire, UK). Nitric acid (Quimex, São Paulo-SP, Brazil) was purified by distillation in a quartz distiller (Quimis). Nitric acid and hydrogen peroxide (Impex, São Paulo, Brazil) were used for sample digestion. A stock solution containing 1000 mg·L^−1^ of Al (Sigma Chemical Corp., St Louis, MO, USA) was used for the preparation of the analytical curve in 0.028 mol·L^−1^ HNO_3_. All measures and procedures were performed in triplicate.

#### 2.4.3. Procedures

All samples were dried using a lyophilizer. Dried samples were ground using a pestle and mortar. Then, the samples were stored in polyethylene containers. A microwave-assisted acid digestion was performed using 4 mL of HNO_3_ and 1 mL of H_2_O_2_ 30% (w/w), both concentrated, for a mass of 0.1 g of sample for the parotid salivary gland and 0.2 g of sample for the submandibular salivary gland. The digested samples were transferred to volumetric flasks and diluted to a final volume of 13.0 mL with deionized water. Blank experiments (*n* = 3) were carried out in the same way. An aliquot of the digested sample was diluted to a final acidity of 0.2% for determination of Al by GFAAS. 

#### 2.4.4. Evaluation of the Accuracy of the Procedure

The accuracy of the procedure was evaluated using the addition method and was based on the analyte recovery. Yields were 125 and 150 µg·L^−1^ Al for the digestion of parotid and submandibular salivary glands, respectively.

### 2.5. Perfusion, Histological Processing and Immunohistochemical Analysis

After collecting the glands on one side for quantification of Al, the animals were transcardially perfused with heparinized 0.9% phosphate-buffered saline (PBS) followed by 4% paraformaldehyde. The contralateral glands were harvested and post-fixed in 6% formaldehyde until final processing. The glands were dehydrated in increasing ethanol solutions (70%, 80%, and 90%) cleared in xylene and embedded in paraplast.

Sections of 5-µm thickness were obtained and mounted on 3-aminopropyltriethoxysilane-coated slides (Sigma Chemical Corp., St Louis, MO, USA). Sections were dewaxed in xylene and hydrated in graded ethanol. Antigen retrieval was carried out with citrate buffer (pH 6.0) in a Pascal chamber (Dako, Carpinteria, CA, USA) for 30 s. Sections were immersed in 3% H_2_O_2_ in methanol for 20 min for inhibition of endogenous peroxidase activity, and then blocked with 1% bovine serum albumin (BSA, Sigma Chemical Corp., St Louis, MO, USA) in phosphate-buffered saline (PBS) for 1 h. Slides were incubated with primary antibodies against cytokeratin 18 (CK-18, 1:100, Millipore, Stockholm, Sweden), α-smooth muscle actin (α-SMA, 1:200, Abcam, Cambridge, UK) and MT I/II (clone E9, 1:50, Dako, Glostrup, Denmark). The primary antibodies were diluted in PBS and incubated for 1 h at room temperature. Subsequently, the sections were incubated for 30 min with biotin-free horseradish peroxidase (HRP) enzyme-labeled polymer (REVEAL, Spring, Pleasanton, CA, USA). Diaminobenzidine (Sigma Chemical Corp., St Louis, USA) was used as the chromagen and sections were counterstained with Mayer’s hematoxylin (Sigma Chemical Corp., St Louis, USA). Replacement of the primary antibodies with non-immune serum served as negative controls.

Brightfield images from at least 5 randomly selected images from each sample were acquired with an Axioskop 40 (Carl Zeiss, Jena, Germany) equipped with a CCD color camera (AxiocCam MRc, Carl Zeiss). All images were acquired at a magnification of 40×. Areas of diaminobenzidine staining were separated and segmented using the color deconvolution plug-in of ImageJ. After image segmentation, the total epithelial glandular staining area was evaluated. The percentage of the area that was stained was calculated and the differences in the percentages among the groups were analyzed.

### 2.6. Statistical Analysis

For the quantification of the Al deposits, descriptive statistics were performed with mean and standard deviation in parts per million (ppm). For the immunohistochemical evaluation, a Mann-Whitney test was performed (*p* < 0.05).

## 3. Results

The results showed deposition of Al in both the parotid and submandibular glands. The parotid glands from the group exposed to Al contained 274.9 ± 0.1 ppm compared to 202.2 ± 0.1 ppm for the control group. The submandibular glands of the group exposed to Al contained 397.3 ± 15.3 ppm compared to 278.7 ± 32.2 ppm for the control group.

**Figure 1:**
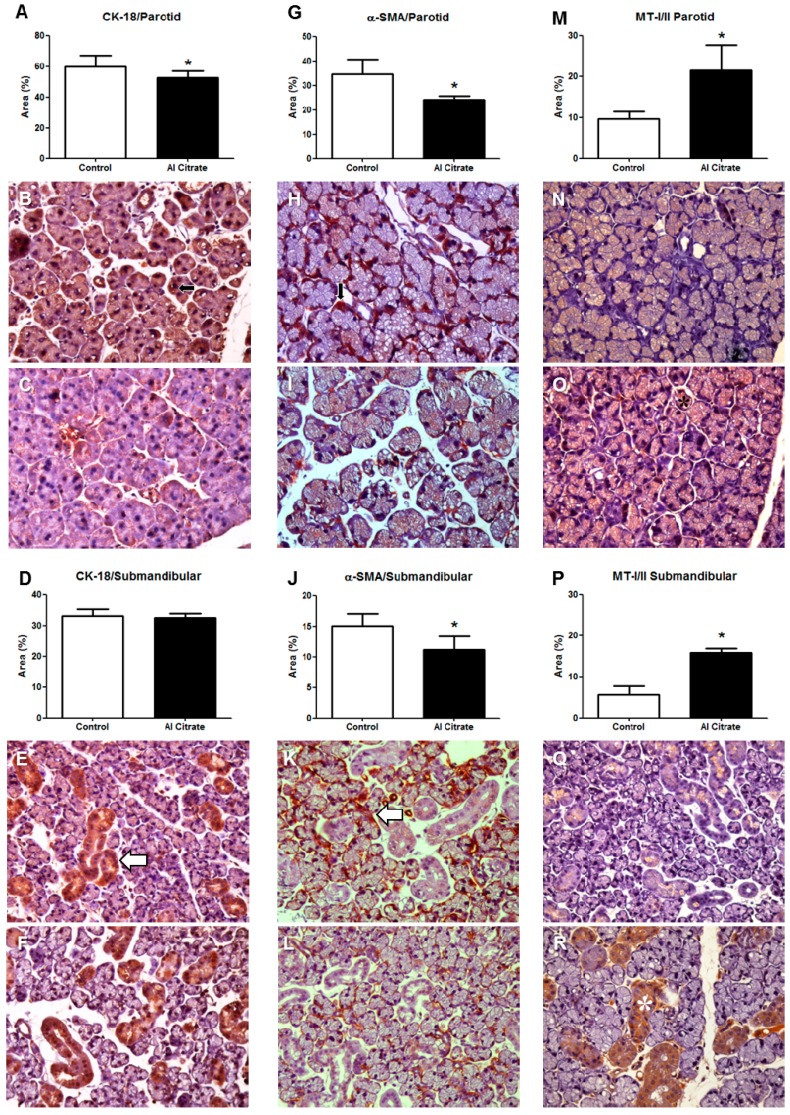
(**A**)**–**(**C**): CK-18 in the parotid; (**D**)**–**(**F**): CK-18 in the submandibular; (**G**)**–**(**I**): α-SMA in the parotid; (**J**)**–**(**L**): α-SMA in the submandibular; (**M**)**–**(**O**): MT-I/I in the parotid; (**P**)**–**(**R**): MT-I/I in the submandibular. (**B**), (**E**), (**H**), (**K**), (**N**), and (**Q**): control. (**C**), (**F**), (**I**), (**L**), (**O**), (**R**): exposed to Al citrate. Arrows and asterisks indicate immunohistochemical staining.

Immunohistochemical evaluation revealed that CK-18 expression decreased in the parotid glands of animals exposed to Al (control = 61.25 ± 1.326, exposed = 50.41 ± 2.175, *p* = 0.0006; Figure 1A–C), while the submandibular gland showed no statistically significant difference (control = 32.69 ± 0.8146, exposed = 29.24 ± 1.957, *p* = 0.1807; Figure 1D–E). 

A decreased expression of α-SMA was observed in the parotid glands of the animals exposed to Al (control = 35.04 ± 1.137, exposed = 23.52 ± 0.8291, *p* = 0.0025; Figure 1G–I). Similar results were found in the glands (control = 14.71 ± 0.7698, exposed = 11.14 ± 0.5696, submandibular *p* = 0.0070; Figure 1J–L). 

The parotid glands of the Al-exposed group showed higher values for MT-I/II (control = 9.150 ± 0.9661, exposed = 19.15 ± 3.310, *p* = 0.0159; Figure 1M–O). Similarly, the submandibular glands of the Al-exposed group had increased MT-I/II expression (control = 5.764 ± 0.6315, exposed = 15.02 ± 1.435, *p* = 0.0357; Figure 1P–R).

## 4. Discussion

In this paper, we provide the first report of the effects of chronic intoxication with Al citrate on the salivary glands of rats. We have measured the Al deposits in these glands, the changes to the cytoskeleton of acinar and myoepithelial cell populations, and an increase in the expression of MT-I/II in the parotid and submandibular glands.

Al toxicity is not fully understood, although there are several works in the literature that have used different methods of intoxication, different doses and various Al compounds to study the effects of this metal in several different organisms [7,21,22].

The transport of Al into the body has yet to be completely elucidated. In mammals, it is known that Al is poorly absorbed from the gastrointestinal tract after ingestion, and that most of the compound is transformed into insoluble salts, mostly AlPO_4,_ in the digestive tract [23,24]. In addition, Al absorption depends on the route of ingestion and this, in turn, depends on the chemical form of the metal [23,25,26], the ability to dissolve gastrointestinal Al, and the acidity of gastric secretions [21,27].

Our choice of Al citrate as the vehicle for intoxication was based on studies reporting that this form of Al crosses the gastrointestinal barrier into the blood [28,29,30,31]. Some authors claim that Al citrate can become soluble Al and a considerable fraction occurs as a neutral complex, capable of easily crossing the membranes and acting as a vehicle that facilitates the absorption of the metal [22,29,30,32,33,34]. One of the hypotheses raised in the literature is that citrate increases bioavailability in the intestine by increasing the permeability of the paracellular channels, possibly by means of a deregulation of calcium homeostasis. This mechanism is unique for the Al citrate complex, which is in line with the high bioavailability of this complex in comparison with other ligand complexes [32,35].

The model of intoxication adopted for this investigation, the administration of 100 mg/kg/day by gavage for 30 days, led to the deposit of Al in the parotid and submandibular salivary glands.

We chose the cytoskeletal components of acinar cells and myoepithelial cells as tissue parameters to assess the effects of Al citrate in salivary glands. In a previous report of intoxication with Al in rats, with a shorter time course and lower doses than those used by us, Denisov [19] was unable to find changes in salivary glands after routine histological processing. Changes in salivary function and composition were observed; however, leading us to the search for more precise tissue parameters to measure.

Cytokeratins (CKs) are the main intermediate filaments of the cytoskeleton of epithelial cells and an indicator of metal toxicity of salivary glands [36]. Normal salivary glands present immunopositivity for all CKs, including CK-18, regardless of the gland component [37]. CK expression patterns differ according to cell type, stage of development, differentiation state, anatomical location, and degree of complexity of the epithelial cells, and is a useful tool for identifying different epithelial types and origins [38].

Traditionally, α-SMA is a myoepithelial cell marker. These cells play an important role in the physiology of the salivary glands and, consequently, in maintaining the health of the oral mucosa. Decreased expression of α-SMA was observed in both parotid and submandibular glands, while CK-18 was reduced only in the parotid gland. The decrease in the expression of these proteins could be responsible for the cytoskeletal damage caused by Al in the glands. There was no difference in the immunoexpression of CK-18 in the submandibular glands of the exposed and control groups. This may suggest that this gland is more resistant to damage by Al, even in the presence of Al deposits.

The oxidative stress produced by intoxication with Al citrate, such as oxidation of proteins, may have contributed to the disorganization of the cytoskeleton, leading to a decreased expression of CK18 in epithelial cells and α-SMA in myoepithelial cells. In normal salivary glands, epithelial cells take part in the production of saliva whereas myoepithelial cells have a contractile action enabling salivary secretion. The results obtained in this study can explain the decrease in the production of saliva observed by Denisov [19].

We also evaluated the expression of MT-I/II. MTs are low molecular weight cysteine-and metal-rich proteins containing sulfur-based metal clusters formed with Zn(II), Cd(II), and Cu(I) ions [39,40]. In mammals, four distinct MT isoforms are found, termed MT-I through MT-IV. The MT-I/MT-II isoforms are widely expressed. Their biosynthesis is induced by a wide range of stimuli, including exposure to metals.

The induction of MTs by exposure to heavy metals has been demonstrated in many tissues, including the liver, kidney, intestine and pancreas [41]. In this work, we demonstrate that Al, at a toxic dose, form of administration, and exposure time, was also capable of stimulating MT-I/II expression in salivary glands.

This protein detoxifies heavy metals, such as mercury and cadmium, as well as promoting the homeostasis of essential metals, including copper and zinc. MT is also considered to be cytoprotective by providing anti-oxidant activity against reactive oxygen species, protecting against DNA damage, promoting cell survival, inhibiting apoptosis, increasing angiogenesis, and increasing cell proliferation [39,42]. Little is known about the expression of MTs when an organism is exposed to Al. Our data suggest that with our intoxication model, the glandular epithelial tissue was able to respond to the damage caused by the Al, such as deposits of this metal and disruption of the cytoskeleton, with an increased expression of MT-I/II, an expression that also occurs after exposure to other metals.

Due to its high sulfhydryl content, MT also contributes to the antioxidant defense activity of the cells, eliminating reactive oxygen species generated by free radicals [43,44]. It is also known that Al mediates oxidative stress [45,46], leading to an increase in reactive oxygen species [47]. The observed expression of MT-I/II may be also associated with a mechanism of cytoprotection, or even with cell survival after intoxication damage.

The harmful effects of Al are known to be neutralized by zinc [48,49], which is an essential metal for cellular function. Zinc is regulated by metallothionein [43]. Al leads to an increase of intracellular zinc concentrations [49]. Therefore, if the zinc content is high there will also be high contents of metallothionein, since this is the protein that regulates zinc entry into the cell, reducing the toxic effects of Al. It is known that metallothionein has a fundamental role in cellular protection against free radicals, preventing lipid peroxidation of the cellular membrane [50]. Al leads to an increase of free radicals in the body [49]. Thus, we expected to observe a direct relation in the increase of metallothionein expression in the salivary glands and intoxication by Al. This increase would occur in an attempt to protect the glandular tissue against the harmful effects of this intoxication.

## 5. Conclusions 

Our results show that Al citrate intoxication promotes Al deposition in glandular tissue. This leads to changes in the cytoskeleton of gland cells (epithelial and myoepithelial), and a cytoprotective increase in the expression of MT-I/II was observed. These findings call for further studies on the mechanisms unleashed by cellular damage and the minimum doses and times of exposure needed to produce injury.
